# Supplementary material for: TssA forms a gp6‐like ring attached to the type VI secretion sheath
Source: EMBO J. 2016 Jun 10;35(15):1613–27. doi: 10.15252/embj.201694024 (PMC4969574; doi:10.15252/embj.201694024)
Supplement: Supplementary file 1 — Appendix [file EMBJ-35-1613-s001.pdf]

Appendix

TssA forms a gp6-like ring attached to the type VI secretion sheath

Sara Planamente[1], Osman Salih[2], Eleni Manoli[1], David Albesa-Jové[3], Paul S. Freemont[2,*] & Alain Filloux[1,**]

[1]MRC Centre for Molecular Bacteriology and Infection (CMBI), Department of Life Sciences, Imperial College London, London SW7 2AZ, United Kingdom.

[2] Section of Structural Biology, Department of Medicine, Imperial College London, London SW7 2AZ, United Kingdom.

[3]Unidad de Biofísica, Consejo Superior de Investigaciones Científicas - Universidad del País Vasco/Euskal Herriko Unibertsitatea (CSIC-UPV/EHU), Departamento de Bioquímica, Universidad del País Vasco, 48940 Leioa, Bizkaia (Spain), and Structural Biology Unit, CIC bioGUNE, Bizkaia Technology Park, 48160 Derio (Spain).

Appendix table of content

Appendix Table S1 and S2

Appendix Figure S1 and S2

Appendix Table S1. Strains used in this study

Strains	Description	References
E. coli		
One-shot top10	F− *mcrA* Δ(*mrr-hsd*RMS-*mcr*BC) φ80*lac*ZΔM15 Δ*lac*X74 *rec*A1 *ara*D139 Δ(*ara-leu*)	Invitrogen
B834(DE3)	F− *ompT hsdS*B(rB− mB−) *gal dcm met* (DE3)	Laboratory collection
DHM1	*cya*-854 *rec*A1 *gyrA96* (NaI) *thi1 hsdR17 spoT1 rfbD1 glnV44*(AS)	Karimova et al., 1998
C118λpir	Host strain for pKNG101 replication; D (*ara-leu*) *araD DlacX74 galE galK-phoA20 thi-1 rpsE rpoB argE* (Am) *recA1 Rf*^{r} (*lpir*)	Herrero et al., 1990
P. aeruginosa		
PAKΔ*retS*	In-frame deletion of *retS* (PA4856) in *P. aeruginosa* PAK wild-type strain	Goodman et al., 2004
PAKΔ*retS*-pBBR	PAKΔ*retS* strain carrying the empty vector pBBR1MCS-4	This study
PAKΔ*retS*Δ*A1*	In-frame deletion of *tssA1* (PA0082) in *PAKΔretS*	This study
PAKΔ*retS*Δ*A1*-pBBR	PAKΔ*retS*Δ*A1*mutant carrying the empty vector pBBR1MCS-4	This study
PAKΔ*retS*Δ*A1*-pBBR-*A1*	PAKΔ*retS*Δ*A1*mutant carrying the pBBR1MCS-4 vector expressing *tssA1* (pBBR-*A1*)	This study
PAKΔ*retS*Δ*A1*-pBBR-*His$_6$A1*	PAKΔ*retS*Δ*A1*mutant carrying the pBBR1MCS-4 vector expressing the N-terminally His_6 tagged *tssA1* (pBBR-*His$_6$A1*)	This study
PAKΔ*retS*Δ*E1*	In-frame deletion of *tssE1* (PA0087) in *PAKΔretS*	Lossi et al., 2011
PAKΔ*retS*Δ*E1*-pBBR	PAKΔ*retS*Δ*E1*mutant carrying the empty vector pBBR1MCS-4	
PAKΔ*retS*Δ*E1*-pBBR-*E1*	PAKΔ*retS*Δ*E1*mutant carrying the pBBR1MCS-4 vector expressing *tssE1* (pBBR-*E1*)	This study
PAKΔ*retS*Δ*B1::B1-sfGFP*	PAKΔ*retS*Δ*B1* strain having the gene encoding the C-terminal fusion of the sfGFP with tssB1 (*B1-sfGFP*) under a plac promoter integrated into the *att* site	This study
PAKΔ*retS*Δ*A1*Δ*B1::B1-sfGFP*	PAKΔ*retS*Δ*A1*Δ*B1* strain having the gene encoding the *B1-sfGFP* under a plac promoter integrated into the *att* site.	This study
PAKΔ*retS*Δ*B1*Δ*E1::B1-sfGFP*	PAKΔ*retS*Δ*B1*Δ*E1* strain having the gene encoding the *B1-sfGFP* under a plac promoter integrated into the *att* site	This study
PAKΔ*retS*Δ*A1*Δ*B1::B1-sfGFP-pBBR-A1*	PAKΔ*retS*Δ*A1*Δ*B1::B1-sfGFP* carrying the pBBR1MCS-4 vector expressing *tssA1* (pBBR-*A1*)	This study
PAKΔ*retS*Δ*B1*Δ*E1::B1-sfGFP-pBBR-E1*	PAKΔ*retS*Δ*B1*Δ*E1::B1-sfGFP* carrying the pBBR1MCS-4 vector expressing *tssE1* (*pBBR-E1*)	This study

Appendix Table S2. Plasmids used in this study

Plasmids	Description	References
pKNG101-Δ*A1*	pKNG101 containing the mutator fragment for deletion of *tssA1*, Str^R	This study
Mini-CTX-*B1-sfGFP*	Mini-CTX-1 encoding *tssB1-sfGFP* under a plac promoter for integration into the *att* site of the *P. aeruginosa* chromosome, Tc^R	This study
pBBR	pBBR1MCS-4, Broad host range vector pBBR1 replicon, Ap^R	Kovach et al., 1995
pBBR-*A1*	pBBR1MCS-4 containing *tssA1*	This study
pBBR-His_6A1	pBBR1MCS-4 containing His_6tssA1	This study
pBBR-*E1*	pBBR1MCS-4 containing *tssE1*	This study
pET28	Expression vector used for expression of N-terminal His_6 tagged proteins, Km^R	Novagen
pET26	Expression vector used for expression of untagged proteins, Km^R	Novagen
pACYC	pACYC-Duet vector, Cm^R	Novagen
pET-His_6A1	pET28 expressing TssA1with an N-terminal His_6 tag (His_6-TssA1)	This study
pET26-*B1C1*	pET26 expressing untagged TssB1C1	This study
pACYC-*A1-B1C1*	pACYC-Duet-1 expressing His_6-tagged TssA1 and TssB1C1 without tag from two separate T7 promoters	This study
pKT25	BTH vector for fusion of target proteins to *B. pertussis cya* gene T25 fragment; P_{lac}::cya^{1-675}p15ori, Km^R	Karimova et al., 1998
pUT18C	BTH vector for fusion of target proteins to *B. pertussis cya* gene T18 fragment; P_{lac}::$cya^{675-1197}$pUCori, Ap^R	Karimova et al., 1998
pKT25-*A1*	Fusion of *tssA1* to *cya* gene T25 fragment in pKT25, Km^R	Lossi et al., 2012
pUT18C-*A1*	Fusion of *tssA1* to *cya* gene T18 fragment in pUT18C, Ap^R	This study
pUT18C-*Hcp1*	Fusion of *hcp1* to *cya* gene T18 fragment in pUT18C, Ap^R	This study
pUT18C-*E1*	Fusion of *tssE1* to *cya* gene T18 fragment in pUT18C, Ap^R	Lossi et al., 2012
pUT18C-*F1*	Fusion of *tssF1* to *cya* gene T18 fragment in pUT18C, Ap^R	Lossi et al., 2012
pUT18C-*G1*	Fusion of *tssG1* to *cya* gene T18 fragment in pUT18C, Ap^R	Lossi et al., 2012
pUT18C-*K1*	Fusion of *tssK1* to *cya* gene T18 fragment in pUT18C, Ap^R	This study
pUT18C-*VgrG1a*	Fusion of *vgrG1a* to *cya* gene T18 fragment in pUT18C, Ap^R	This study
pUT18C-*ClpV1*	Fusion of *ClpV1* to *cya* gene T18 fragment in pUT18C, Ap^R	Förster et al., 2014

Ap^R, ampicillin-resistant; Cm^R, chloramphenicol-resistant; Km^R, kanamycin-resistant; Str^R, streptomycin-resistant; Tc^R, tetracyclin-resistant.

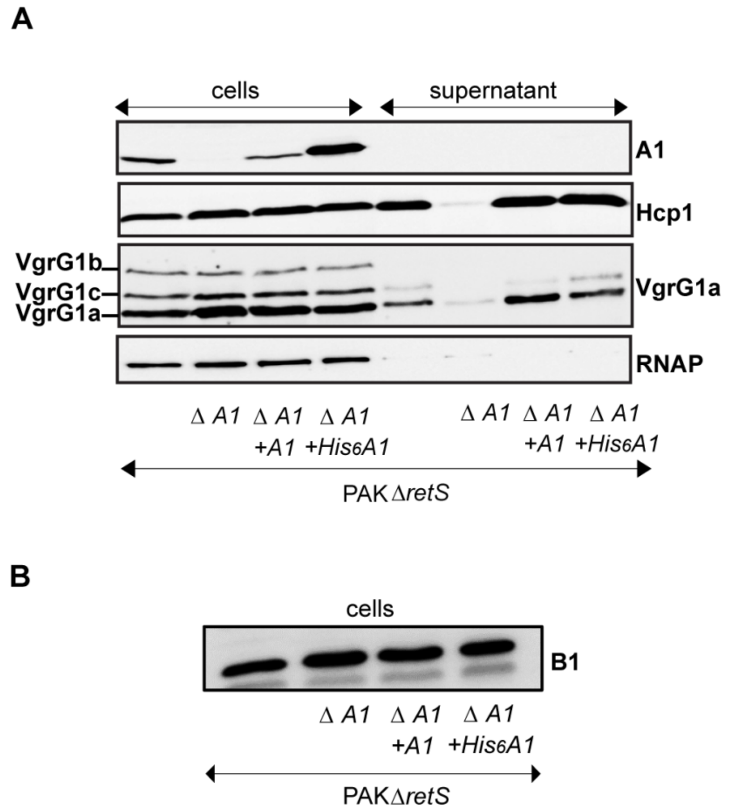

Appendix Figure S1. The N-terminally His$_6$-tagged TssA1 is functional. A. Proteins from whole cell extracts and culture supernatants of PAKΔ*retS*, the derivative *tssA1* mutant (Δ*A1*) (both carrying the pBBR plasmid) and the complemented Δ*A1* mutant carrying pBBR-*tssA1* (Δ*A1*+*A1*) or pBBR-*His$_6$-tssA1*(ΔA1+His$_6$A1) were analysed by Western blotting. Polyclonal antibodies directed against TssA1 (A1), Hcp1 and VgrG1a were used. The anti-VgrG1a antibody detects VgrG1a, VgrG1b and VgrG1c, as indicated on the left. RNA polymerase (RNAP) was monitored in both whole-cell lysates and culture supernatants using monoclonal antibody directed against its β-subunit. **B.** Western blot analysis showing the production of TssB1 in PAKΔ*retS*, Δ*A1* (both carrying the pBBR plasmid) and the complemented strains Δ*A1*+*A1* and Δ*A1*+*His$_6$A1*. Polyclonal antibody directed against TssB1 was used.

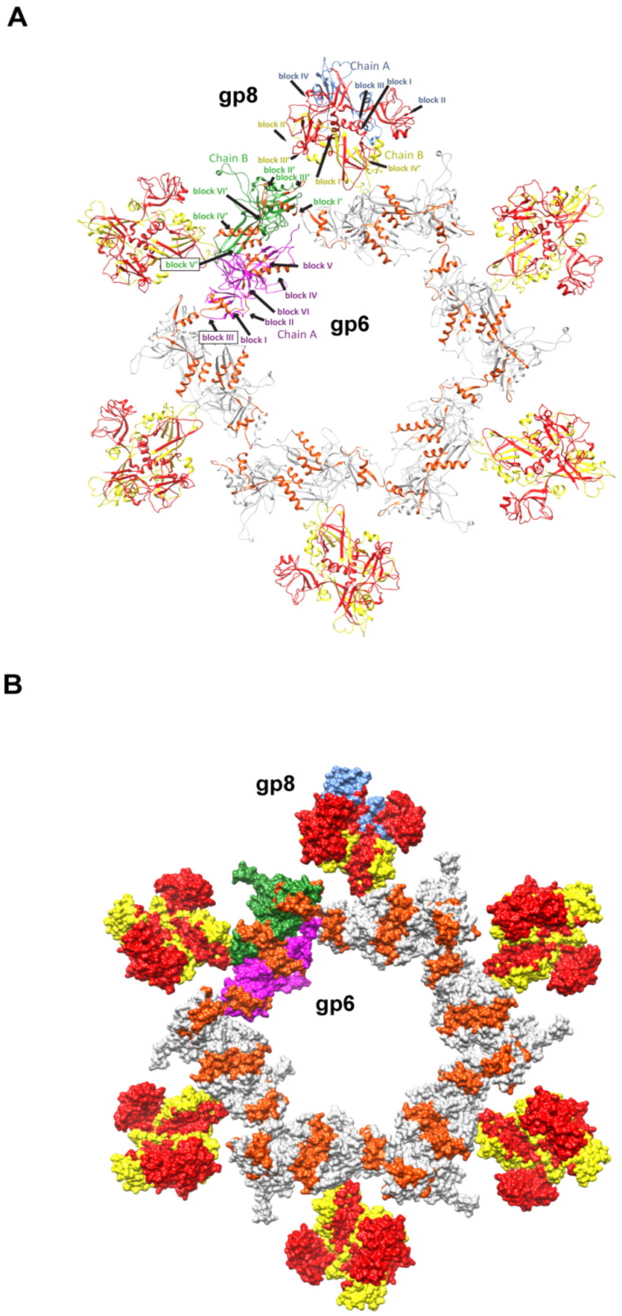

Appendix Figure S2. The gp6/gp8 baseplate subcomplex of the T4 phage. (A), Cartoon and **(B),** surface representations of the dodecameric gp6 structure (PDB code 3H3W) and the structure of gp8 (PDB code 1PDM) are shown. In both representations, chain A of the gp6 dimers is shown in magenta and chain B in green and the TssA1/gp6 conserved regions are shown in orange in the gp6 ring. The TssA1/gp8 conserved blocks are shown in red in the 3D structure of gp8. The conservation is most significant in regions involved in gp8 dimerization and interaction with gp6.
