# Supplementary material for: TssA forms a gp6‐like ring attached to the type VI secretion sheath
Source: EMBO J. 2016 Jun 10;35(15):1613–27. doi: 10.15252/embj.201694024 (PMC4969574; doi:10.15252/embj.201694024)
Supplement: Supplementary file 2 — Expanded View Figures PDF [file EMBJ-35-1613-s002.pdf]

Expanded View Figures

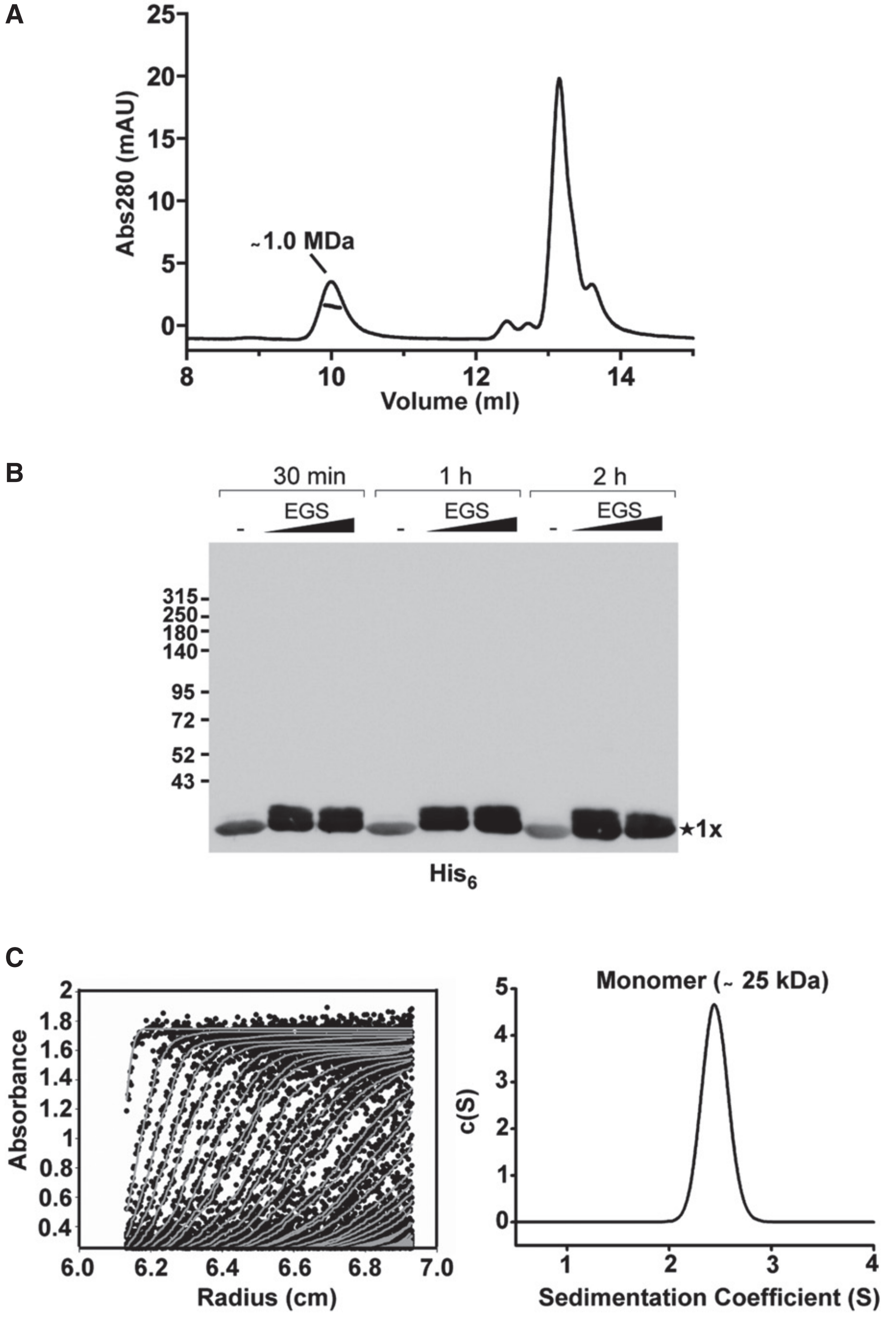

Figure EV1. Removal of the C-terminal moiety of TssA1 leads to its monomeric form.

A SEC-MALS analysis of the purified His_6-TssA1. Experiments were carried out on an Agilent Technologies 1200 Series HPLC using a WYATT 100S5 column coupled to a WYATT MALS instrument. The eluted peaks of His_6-TssA1 and the corresponding mass are shown.

B Western blot showing *in vitro* cross-linking experiments using the purified C-terminally truncated protein $TssA1_{1–245}$. About 30 μg of purified His_6-$TssA1_{1–245}$ was cross-linked (30 min, 1 and 2 h) at room temperature using increasing amounts of EGS (2 and 5 mM) where indicated. Samples were analysed by 3–12% gradient SDS–PAGE and $TssA1_{1–245}$ immunodetected using an anti-His_6 monoclonal antibody.

C Sedimentation data of His_6-$TssA1_{1–245}$ (1.6 mg/ml) recorded at a rotor speed of 50,000 rpm are shown. The left panel shows the sedimentation boundary fits. For clarity, only every third scan is shown in the fitted data plots where the experimental absorbance data are shown as black circles, whereas the boundary fits are shown as grey lines. The right panel shows the size-distribution analysis c(s), obtained from fitting the scan boundaries using SEDFIT, revealing a monomeric peak (Mr ~25 kDa) at $S_{20,w}$ value of 2.4 S.

© 2016 The Authors

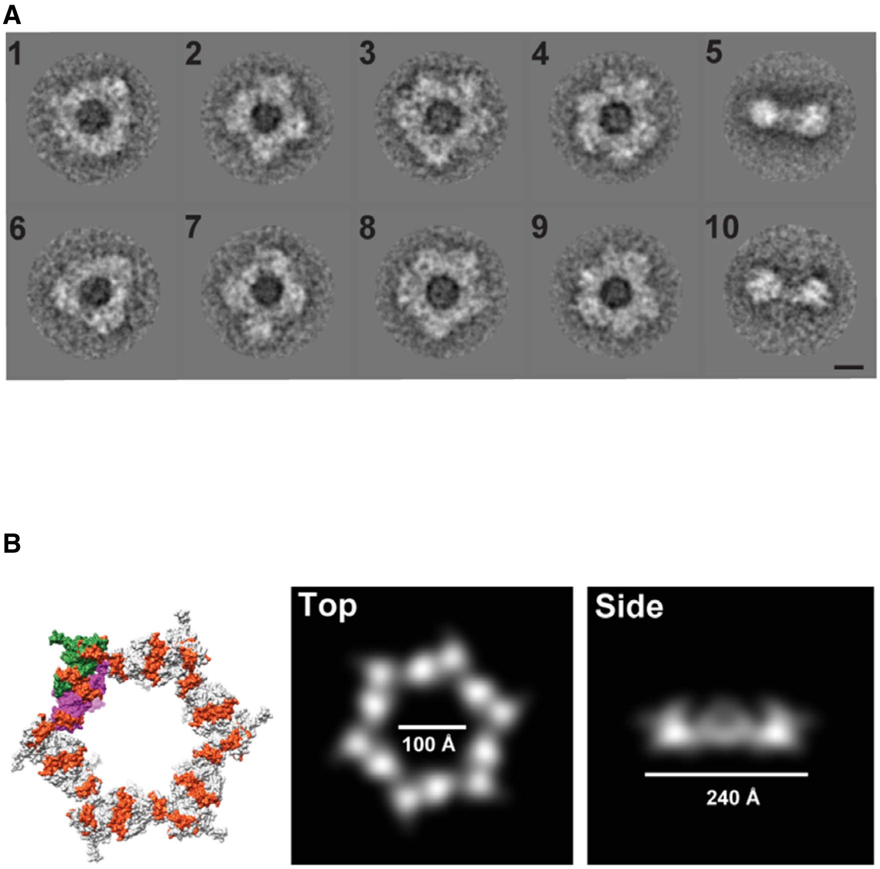

Figure EV2. 2D classification of TssA1 rings using IMAGIC and re-projections of the gp6_334C ring.

A Representative class averages of the TssA1 complex obtained using IMAGIC-5. The classification shows the wide range of conformations taken by the ring-shaped structures, ranging from a threefold-like symmetry (classes 1 and 6) to a sixfold symmetry-like (classes 4 and 9). Scale bar is 100 Å.

B Surface representations of the dodecameric gp6 structure (PDB code 3H3W) with conserved blocks indicated in orange. Chain A of gp6 dimers is shown in magenta and chain B in green (left). Re-projection images of gp6_334C (PDB code 3H3W) filtered to 60 Å resolution shown in top and side views (right panels). The dodecameric ring structure of T4 phage gp6_334C has dimensions comparable to those measured for the T6SS TssA1 complex. The re-projected image of gp6_334C has a diameter of about 240 Å, an inner hole of ~100 Å and a width of ~80 Å. The TssA1 ring has a similar diameter of about 260 Å, an inner hole of ~100 Å and a width of ~95 Å.

© 2016 The Authors

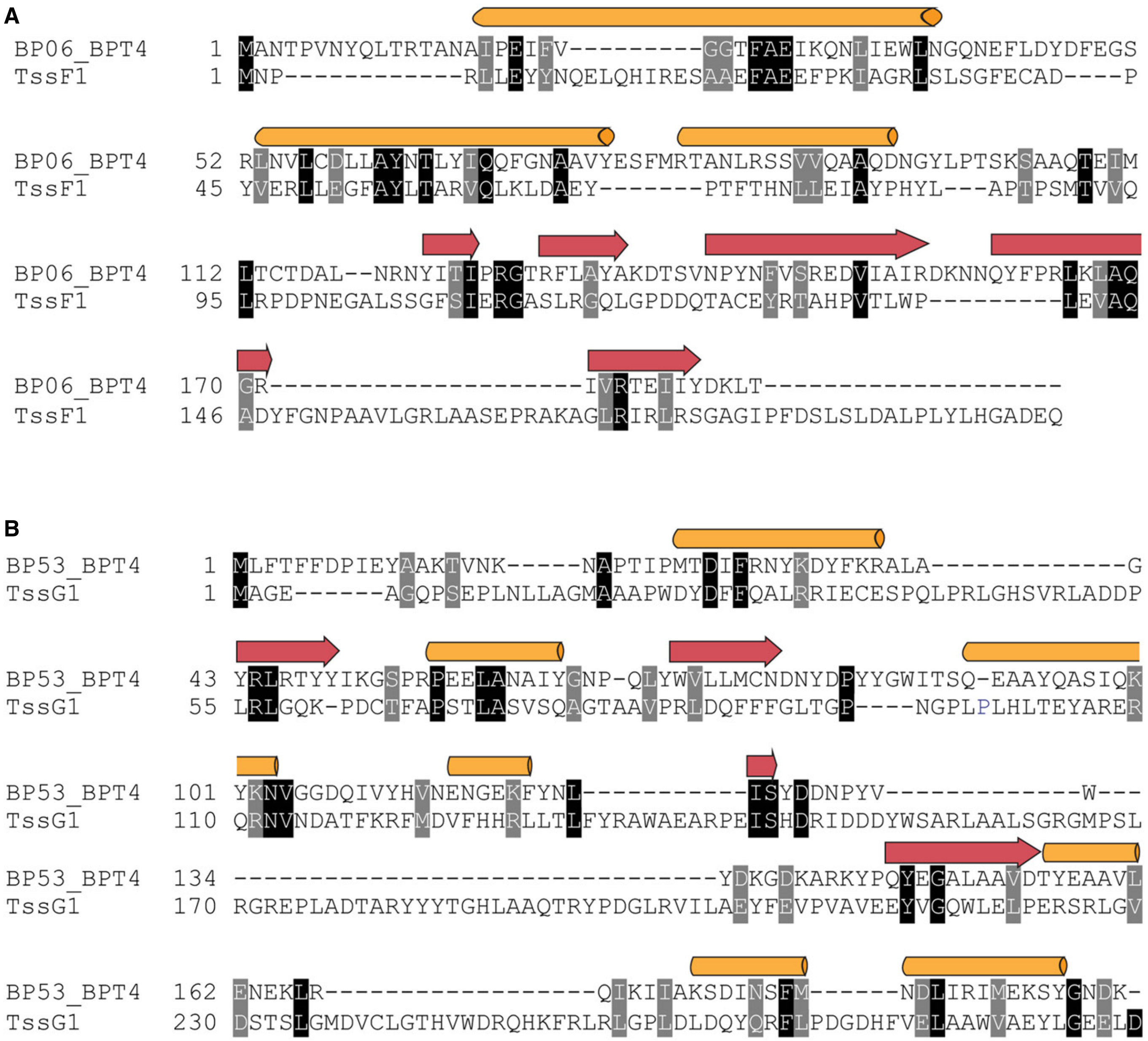

Figure EV3. Secondary-structure-weighted sequence alignment of TssF1 with the N-terminal region of gp6 and TssG1 with gp53.

A, B Protein sequences were extracted from UniProt accession numbers BP06_BPT4 and BP53_BPT4 for enterobacteria phage T4 (gp6 and gp53, respectively; bacteriophage T4) and the *Pseudomonas* Genome Database (TssF1 and TssG1; reference strain PAO1). Conserved positions from (A) the TssF1/gp6 and (B) the TssG1/gp53 sequence alignments are shown in black and grey background. Secondary-structure-weighted sequence alignments were carried out using PROMALS3D.

Figure EV4. The TagJ-like region of EcTssA/TssA2.

A On the left is shown the superposition of the ribbon structures of PaTagJ (PDB code 4UQZ, yellow), the C-terminal region of *Ec*TssA (PDB code 4YO5, blue) and a 3D structural model of the *Pa*TssA2 C-terminal region (red). The right panel shows the ribbon structures of *Pa*TagJ (yellow), Cter *Ec*TssA (blue) and *Pa*TssA2 model (red).

B Sequence alignment of N-terminal TagJ and C-terminal regions of *Ec*TssA and *Pa*TssA2. The 3D structural model of *Pa*TssA2 has been predicted using Phyre2, who found *Ec*TssA as the highest homologous structure with a 22% sequence identity and 98% confidence score.

C BTH experiment showing interaction between TssA1 (A1) and TagJ. A graphical representation of β-galactosidase activity from *E. coli* DHM1 cells producing the indicated proteins fused to the adenylate cyclase T25 or T18 subunits is shown.

 © 2016 The Authors

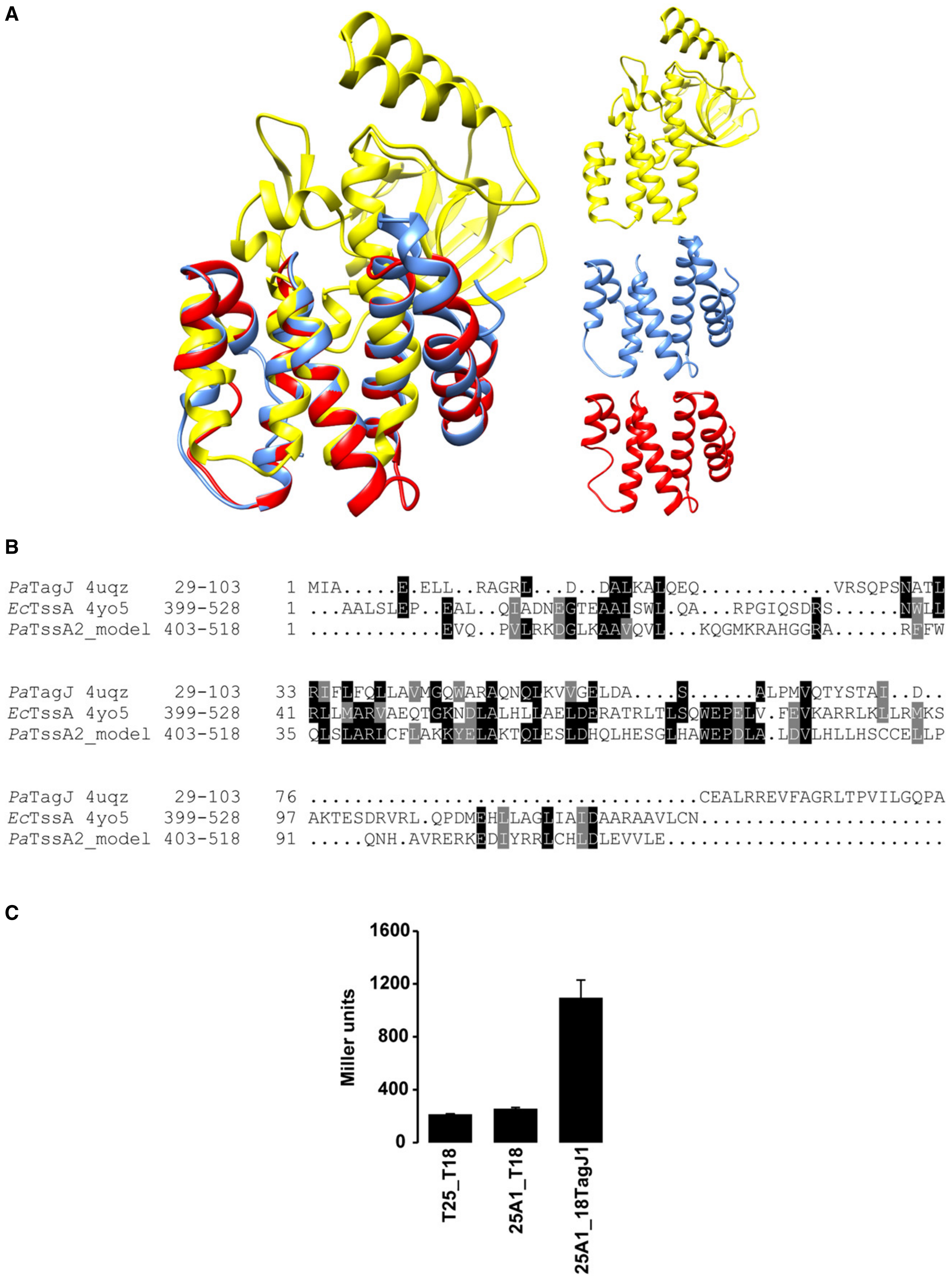
A
B
PaTagJ 4uqz 29-103 1 MIA.....E.ELL..RAGRL...D..DALKALQEQ............VRSQPSNATL
EcTssA 4yo5 399-528 1 ...AALSLEP..EAL..QIADNEGTEAALSWL.QA...RPGIQSDRS......NWLL
PaTssA2_model 403-518 1 ...........EVQ..PVLRKDGLKAAVQVL...KQGMKRAHGGRA......RFFW
PaTagJ 4uqz 29-103 33 RIFLFQLLAVMGQWARAQNQLKVVGELDA....S......ALPMVQTYSTAI..D..
EcTssA 4yo5 399-528 41 RLLMARVAEQTGKNDLALHLLAELDERATRLTLSQWEPELV.FEVKARRLKLLRMKS
PaTssA2_model 403-518 35 QLSLARLCFLAKKYELAKTQLESLDHQLHESGLHAWEPDLA.LDVLHLLHSCCELLP
PaTagJ 4uqz 29-103 76 .................................CEALRREVFAGRLTPVILGQPA
EcTssA 4yo5 399-528 97 AKTESDRVRL.QPDMEHILAGLIAIDAARAAVLCN.....................
PaTssA2_model 403-518 91 .....QNH.AVRERKEDIYRRLCHLDLEVVLE........................
C
Miller units
1600
1200
800
400
0
T25_T18
25A1_T18
25A1_18TagJ1

Figure EV4.

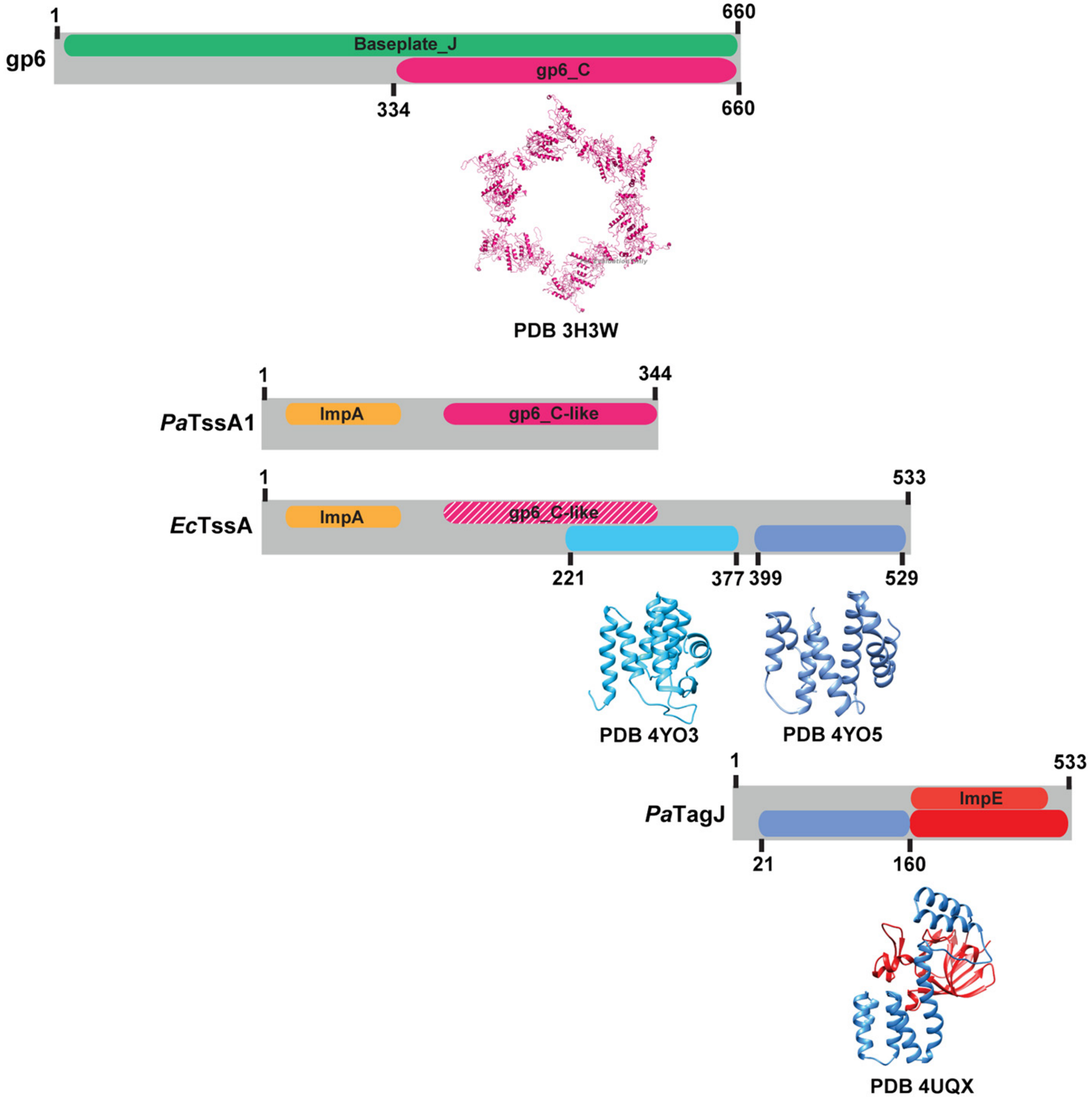

Figure EV5. Comparison between gp6, TssA proteins and TagJ.

Schematic representation of similarities/differences between gp6, *Pa*TssA1, *Ec*TssA and TagJ described in this study. For clarity, the amino acid sequences of these proteins are represented as grey blocks with the corresponding name indicated on the left. Within the grey blocks, coloured boxes indicate the superfamily annotation (top) and regions of known 3D structure (bottom). The ribbon structure and the PDB code are shown below the corresponding colour box. The gp6_C-like region found in *Ec*TssA is represented as dashed pink box in order to highlight the weaker conservation of this region in this protein. Numbers shown indicate amino acid position.

© 2016 The Authors
